# Microwave-Assisted In Situ Synthesis of NiMn_2_O_4_ Nanoparticles Embedded in NiCo_2_O_4_ Nanosheets on Nickel Foam as Binder-Free Electrode Material for High-Performance Supercapacitors

**DOI:** 10.3390/nano16120752

**Published:** 2026-06-15

**Authors:** Shusen Wang, Xiaomei Du, Yingqing Fu, Liu Yang, Naibao Huang, Tianxiang Peng

**Affiliations:** 1 College of Public Security Management, LiaoNing Police College, Dalian 116036, China; 2 Materials Sciences & Engineering, Dalian Maritime University, Dalian 116026, China

**Keywords:** NiCo_2_O_4_, NiMn_2_O_4_, electrochemical performance, supercapacitor

## Abstract

Binder-free NiMn_2_O_4_@NiCo_2_O_4_ nanocomposites with NiMn_2_O_4_ nanoparticle (NP) surface coverage on NiCo_2_O_4_ nanosheets (NSs) are fabricated on nickel foam (NF) via a two-step microwave-assisted hydrothermal (MAH) method combined with annealing treatment, which can be used as a high-performance electrode material for supercapacitors. Specifically, a tulle-like NiCo_2_O_4_ nanosheet framework is first in situ grown on NF, followed by the growth of NiMn_2_O_4_ NPs on the surface of NiCo_2_O_4_ NSs via a secondary MAH process. To investigate the effect of the second-step holding time (HT) of MAH on material performance, a series of experiments were carried out with an HT of 15, 30, 45, and 60 min, and the microstructures and electrochemical properties of the products were analyzed. Structural characterization results confirm the successful synthesis of well-defined NiMn_2_O_4_-NPs@NiCo_2_O_4_-NSs composites. Electrochemical tests demonstrate that the product at an HT of 30 min has the best electrochemical performance with a higher specific capacitance of 441.56 F·cm^−2^ at 1 A·cm^−2^ and cycling stability (75% capacitance retention after 5000 cycles at 15 A·cm^−2^). The superior electrochemical properties are mainly attributed to the unique porous tulle-like NS structure with the largest specific surface area of the 30 min product. This distinctive structure affords abundant electrochemical active sites, effectively prevents structural collapse during long-term cycling, and shortens the transmission and diffusion pathways of electrons and electrolyte ions. The optimized NiMn_2_O_4_@NiCo_2_O_4_ electrode material presents extensive application prospects for high-performance supercapacitors.

## 1. Introduction

In order to mitigate global energy crisis and environmental pollution, extensive research efforts have been devoted to exploring renewable energy resources and developing high-efficiency green energy storage and conversion device [1,2]. As a new type of green energy storage device, a supercapacitor (SC) can offer a promising alternative approach for increasing power demands and is worthy of further research and development, due to its advantages of high power density and long cycle life [3,4]. Electrode materials play a dominant role in the performance and efficiency of SCs [5,6], among which transition metal oxides and their composites are widely used for their high theoretical specific capacity, such as MnO [7], NiO [8], CoO [9,10], etc. Compared to single-component metal oxides, multi-metal oxides have attracted increasing attentions in recent years. Their enhanced capacitive performance is primarily derived from the synergistic electrochemical effects between different metal ions, which effectively overcome the performance limitations of single-phase materials [11,12]. Notably, both NiCo_2_O_4_ and NiMn_2_O_4_ show an AB_2_O_4_ spinel structure and excellent electrochemical activities with many advantages of good structural stability, higher electrical conductivity and reversibility. To date, numerous studies have reported on its applications in SCs [13,14,15,16,17,18,19]. For example, S. J. Kim and co-workers [13] synthesized NiCo_2_O_4_ NSs via a hydrothermal method using cobalt nitrate and nickel nitrate precursors at 95 °C for 10 h and then further calcined at 400 °C for 2 h. The NiCo_2_O_4_ NSs electrode presents a high specific capacitance of 332 F·g^−1^ and capacity retention of 86% after 2000 cycles at 2.5 A·g^−1^. N. Huang et al. [16] employed a microwave-assisted hydrothermal method to self-assemble spinel NiMn_2_O_4_ microspheres composed of nanosheets on 3D nickel foam. The prepared NiMn_2_O_4_ exhibited high specific capacitance up to 768.9 F·g^−1^ at 1 A·g^−1^ and outstanding cyclic stability of 86% capacity retention after 6000 cycles at 5 A·g^−1^. L. Lin et al. [19] first constructed NiCo_2_O_4_ nanoneedle@NiMn_2_O_4_ NS core–shell arrays on carbon fabric (CF). Due to the core–shell microstructure, the CF@NiCo_2_O_4_@NiMn_2_O_4_ electrode displays a very high specific capacitance of 539.2 F·g^−1^ at 2 A·g^−1^, and a capacitance retention of 93.0% even after 5000 cycles at 5 A·cm^−2^. A series of similar studies [18,20,21,22,23] demonstrated that constructing nanocomposites composed of two kinds of different oxides with reasonable three-dimensional (3D) structures is a better strategy for meliorating the comprehensive electrochemical performance.

In this work, a well-designed NiCo_2_O_4_ based nanocomposite is successfully fabricated via a facile two-step microwave-assisted hydrothermal (MAH) strategy, aiming to simultaneously improve the energy density, power density, and long-term cycling stability of supercapacitor electrodes. Specifically, three-dimensional spinel NiCo_2_O_4_ nanosheet (NS) arrays were first in situ grown on nickel foam (NF). Subsequently, NiMn_2_O_4_ NPs were synthesized on the NiCo_2_O_4_ NSs by microwave-assisted hydrothermal method. The influence of the microwave-assisted hydrothermal holding time (MAHHT) on the structure and electrochemical properties of the product was investigated. A new structure was constructed, in which NiCo_2_O_4_ NSs are coated on a NiMn_2_O_4_ NP surface. The resulting NiMn_2_O_4_@NiCo_2_O_4_ nanocomposites are conducive to electrochemical reactions and can be used as an electrode for SCs. When the MAHHT is 30 min, the product morphology is NiCo_2_O_4_ tulle-like NS surface coverage on NiMn_2_O_4_ NPs, and its electrochemical performance is the best. It exhibits a high specific capacitance of 441.56 F·cm^−2^ at 1 A·cm^−2^ and good cyclic stability with a capacity retention of 75% after 5000 cycles at 15 A·cm^−2^.

## 2. Experimental Method

### 2.1. Material Preparation

#### 2.1.1. Pretreatment of NF

The NF substrate (Aiblue High-Tech Materials (Dalian) Co., Ltd., Dalian, China) was cut into rectangular pieces of 2 × 3 cm^2^, which were soaked in 3 M dilute hydrochloric acid and ultrasonically treated for 3 to 5 min to remove the oxide layer on the NF surface until a few NF chips appeared in the solution. The dilute hydrochloric acid was poured into the beaker, and the NF was rinsed several times with deionized water to avoid residual hydrochloric acid. Then the NF was ultrasonically cleaned with absolute ethanol for 2 to 3 min, and taken out and placed in an air-blast drying oven to dry at 60 °C for 1 h.

#### 2.1.2. Synthesis of NiCo_2_O_4_ NSs on NF

**Figure 1:**
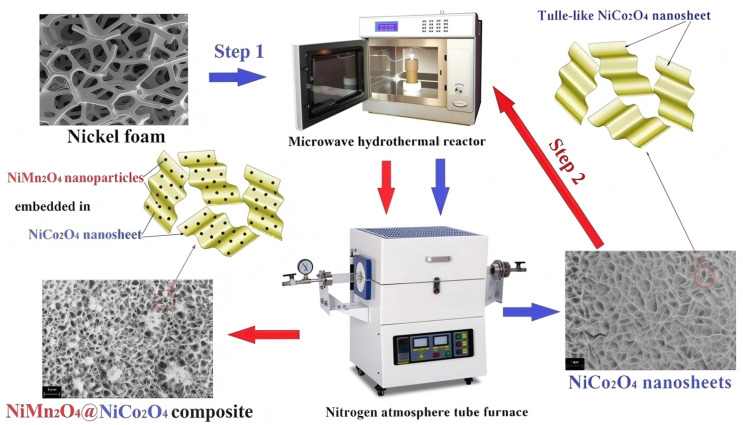
Schematic illustration of the synthetic process for NiCo_2_O_4_ NSs surface coverage on NiMn2O4 NPs. 199 × 115mm (300 × 300 DPI).

The NiCo_2_O_4_ NSs on NF were prepared by a microwave-assisted hydrothermal method combined with an annealing process, as shown in Figure 1 (marked by “Step 1” and blue arrows). At first, 1.041 g Ni (NO_3_)_2_·6H_2_O (Tianjin Damao Chemical Reagent Factory, Tianjin, China) and 2.084 g Co (NO_3_)_2_·6H_2_O (Shanghai Aladdin Biochemical Technology Co., Ltd., Shanghai, China) were added to 216 mL methanol under magnetic stirring for 20 min to form a homogeneous solution. Then 7.160 g hexamethylenetetramine was added in the solution and magnetically stirred until the mixed solution became clear. A piece of pretreated NF substrate was immersed in the mixed solution for 30 min. Then the 35 mL mixed solution and fully soaked NF substrate were diverted into a 100 mL Teflon-lined microwave autoclave, and the microwave autoclave was put into a microwave hydrothermal reactor (XH-800 G, Beijing Xianghu Science and Technology Development Co., Ltd., Bejing, China) heated at 70 °C for 10 min with the power set to 800 W. After the reaction, the microwave autoclave was naturally cooled to room temperature, and the NF sample was taken out, followed by ultrasonic cleaning with deionized water and absolute ethanol several times. The sample was air-blast dried at 60 °C for 12 h and then annealed at 300 °C (5 °C/min heating rate) for 4 h in a nitrogen atmosphere. After natural cooling to room temperature, the sample of NiCo_2_O_4_ NSs in situ grown on NF can be obtained. All the other reagents all bought from Tianjin Damao chemical reagent factory.

#### 2.1.3. Synthesis of NiMn_2_O_4_ NPs on NiCo_2_O_4_ NSs

As marked by “Step 2” and red arrows in Figure 1, the NiMn_2_O_4_ NPs on NiCo_2_O_4_ NSs were synthesized by a method similar to that of growing NiCo_2_O_4_ NSs on NF mentioned above. Firstly, 0.0582 g Ni (NO_3_)_2_·6H_2_O, 0.0444 g NH_4_F and 0.1800 g CO (NH_2_)_2_ were added to 30 mL deionized water under magnetic stirring for about 15 min to get a light-green clear solution. Secondly, 0.0632 g KMnO_4_ was added into the solution under continuous magnetic stirring for 20 min. Then a piece of the NF sample with in situ grown NiCo_2_O_4_ NSs (just mentioned) was immersed in the mixed solution for 30 min. After that, the soaked NF sample and the mixed solution were transferred into a 100 mL Teflon-lined microwave autoclave. The microwave autoclave was put into a microwave hydrothermal reactor (XH-800 G) heated at 160 °C for [15], and 60 min respectively, with the power set to 1000 W. After natural cooling to room temperature, the NF samples were taken out and cleaned ultrasonically with deionized water and ethanol several times to remove residual ions. The above samples were air-blast dried at 60 °C for 12 h. Finally, the samples were annealed at 450 °C (10 °C/min heating rate) for 2 h in a nitrogen atmosphere, and then naturally cooled to room temperature, to get the nanocomposites of tulle-like NiCo_2_O_4_ NSs surface coverage on NiMn_2_O_4_ NPs on NF.

### 2.2. Material Characterization

The crystalline structure of synthesized sample was analyzed by X-ray diffraction (Japan Rigaku, DMAX- Ultima+ diffraction meter, Tokyo, Japan) using Co Kα radiation (λ = 1.7902 Å). The morphology and atomic content were characterized by field emission scanning electron microscopy (FE-SEM), high-resolution transmission electron microscopy (HRTEM) and energy dispersive X-ray spectroscopy (EDS). The composition of elemental and its chemical valence states were investigated by X-ray photoelectron spectroscopy (XPS, USA THERMO ESCALAB250 system (Waltham, MA, USA), Al Ka radiation).

### 2.3. Electrochemical Measurement

The sample’s electrochemical performance was measured on the VMP3 (EG&G) electrochemical workstation in a 6 M KOH solution using a three-electrode system, i.e., the obtained NF sample used as a working electrode. The capacitance calculations are based only on the deposited active material. A platinum net was used as a counter electrode and a saturated calomel electrode (SCE) was used as a reference electrode. The potential window of cyclic voltammetry (CV) was carried out from −0.3 to 0.5 V with a scan rate of 10, 20, 50 and 100 mV·s^−1^, respectively. The galvanostatic charge/discharge (GCD) tests were performed at a current density of 1, 2, 5 and 10 A·g^−1^, respectively. The cyclic charge/discharge (CCD) test was performed at the current density of 10 A·g^−1^. The specific capacitance of the synthesized samples was calculated by CV and GCD curves using Equations (1) and (2), respectively.

(1) $$C=\frac{\int_{V1}^{V2}idV}{2mv∆V}$$

(2) $$C=\frac{i\cdot ∆t}{m\cdot ∆V}$$

where *C* represents the specific capacitance (F·g^−1^); *i* is the current (A); *m* shows the mass (g); *v* is the potential scan rate (V·s^−1^); ∆*V* (*V_2_* ―*V_1_*) is the potential window (V); and ∆*t* is discharge time (s). The measured area of NF immersed in the electrolyte was about 1 × 1 cm^2^.

## 3. Results and Discussion

### 3.1. Structure and Electrochemical Performance of NiCo_2_O_4_ NSs

**Figure 2:**
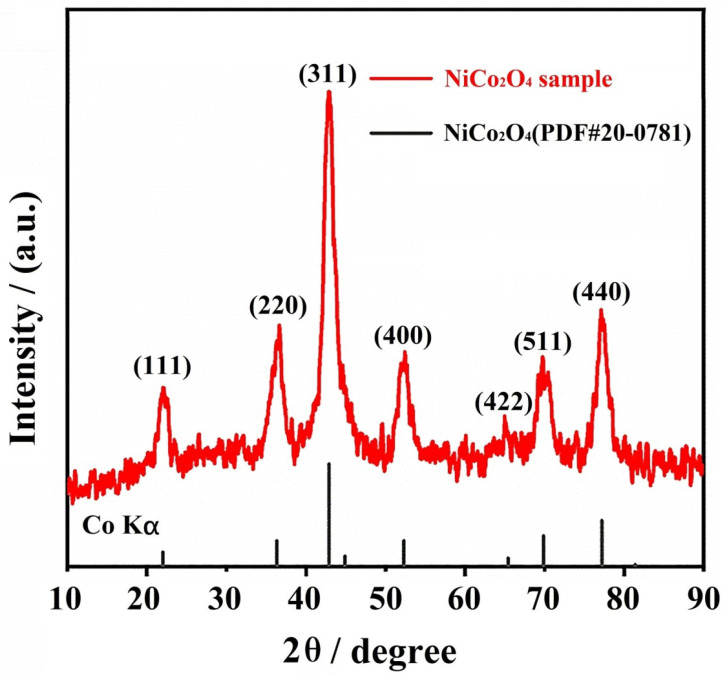
XRD pattern of the NiCo_2_O_4_ sample prepared by the microwave hydrothermal method; (300 × 300 DPI).

**Figure 3:**
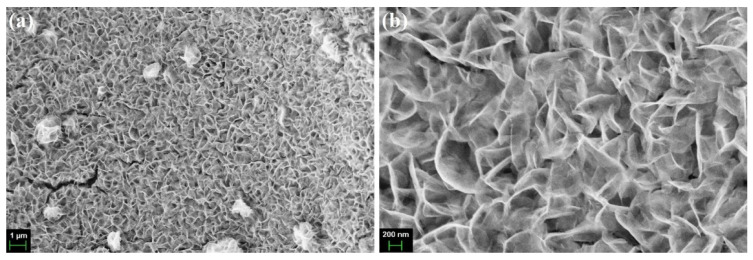
FESEM images of the NiCo_2_O_4_ sample prepared by the microwave hydrothermal method (**a**) Low-magnification image (10 K) (**b**) High-magnification image (50 K); (300 × 300 DPI).

**Figure 4:**
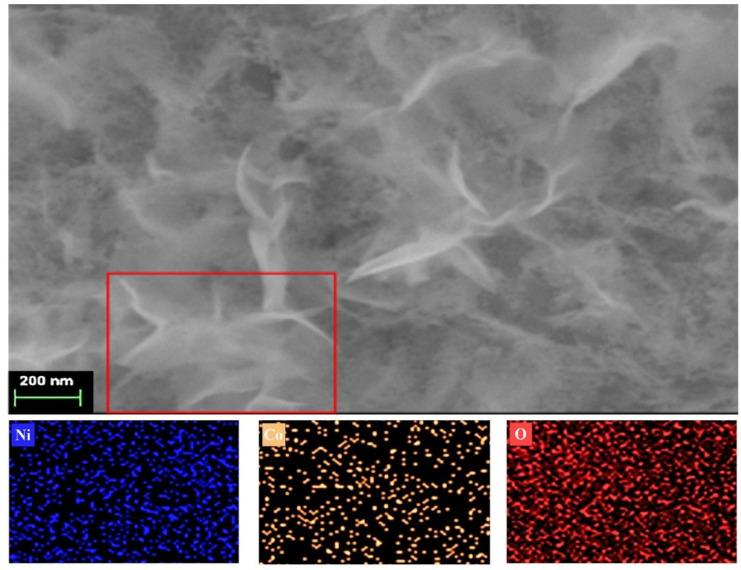
EDS elemental mapping of the NiCo_2_O_4_ sample prepared by the microwave hydrothermal method; 108 × 83 (300 × 300 DPI).

Figure 2 shows the XRD pattern of the NiCo_2_O_4_ sample. It can be clearly seen that the diffraction peaks are indexed at 2θ values of 21.99, 36.33, 42.88, 52.32, 65.39, 69.87 and 77.18 degree, corresponding to the (111), (220), (311), (400), (422), (511) and (440) crystal planes of NiCo_2_O_4_ (PDF#20-0781), respectively. This reveals that NiCo_2_O_4_ is successfully synthesized on the surface of the 3D framework of the NF substrate. The micromorphology of the NiCo_2_O_4_ sample was observed using FE-SEM, as shown in Figure 3. The corresponding EDS elemental mappings (Figure 4) including the elements Ni, Co, and O prove that the three elements are uniformly distributed. Combined with the XRD analysis above, the EDS mapping results further confirm the existence of NiCo_2_O_4_ NSs growing directly on the surface of the NF substrate.

As shown in Figure 3a, vertically aligned petal-like NiCo_2_O_4_ NS arrays are uniformly grown on the nickel foam (NF) substrate to form an interconnected network architecture. This well-distributed NiCo_2_O_4_ nanoarray structure can effectively enlarge the interfacial contact area between the electrode and electrolyte, facilitate electrolyte penetration, and further accelerate the redox reaction kinetics. The magnified FE-SEM image (Figure 3b) further reveals that the mutually staggered NiCo_2_O_4_ NSs with a smooth surface are ultra-thin and their average thickness and length of the side are about 5–10 nm and 350 nm, respectively. Clearly, such microstructure benefits the fast transfer of electrolyte ions during electrochemical reaction, gives NiCo_2_O_4_ a larger specific surface area, and also makes it feasible to recombine with NiMn_2_O_4_ afterwards.

From the EDS images in Figure 4, it can be observed that the Ni, Co, and O elements are uniformly distributed in the nanosheet area.

**Figure 5:**
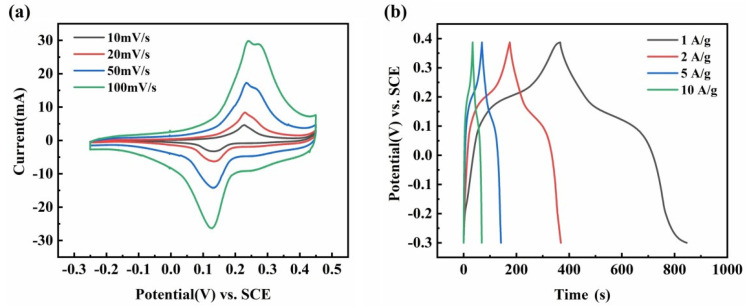
(**a**) CV curves of the NiCo_2_O_4_ sample prepared by the microwave hydrothermal method at different scan rates (10, 20, 50, and 100 mV· s^−1^, respectively). (**b**) GCD curves of the NiCo_2_O_4_ sample prepared by the microwave hydrothermal method under different current densities (1, 2, 5, and 10 A·g^−1^ respectively); (300 × 300 DPI).

Figure 5a shows the CV curves of the NiCo_2_O_4_ sample measured within an identical potential window (10, 20, 50, and 100 mV·s^−1^, respectively). Obvious redox peaks can be seen in the CV curves, showing good pseudocapacitance characteristics. As the scan rate increases, the curve does not deform significantly, and the positions of the redox peaks remain almost the same, indicating that the sample is not greatly affected by polarization and has good reversibility and magnification. Figure 5b shows the GCD curves of the sample under different current densities (1, 2, 5, and 10 A·g^−1^), respectively. The apparent voltage platforms observed during the charge and discharge processes further verify the pseudocapacitive behavior of the NiCo_2_O_4_ electrode. According to the calculation based on Equation (2), the specific capacitances of the sample are 698.655, 563.708, 524.567, and 505.381 F·g^−1^ at current densities of 1, 2, 5, and 10 A·g^−1^, respectively. This indicates that the prepared NiCo_2_O_4_ sample has high specific capacitance and good rate performance, and it can be expected to have more excellent performance after being composited with NiMn_2_O_4_ subsequently.

### 3.2. Structure and Electrochemical Performance of NiMn_2_O_4_@NiCo_2_O_4_ Composites

**Figure 6:**
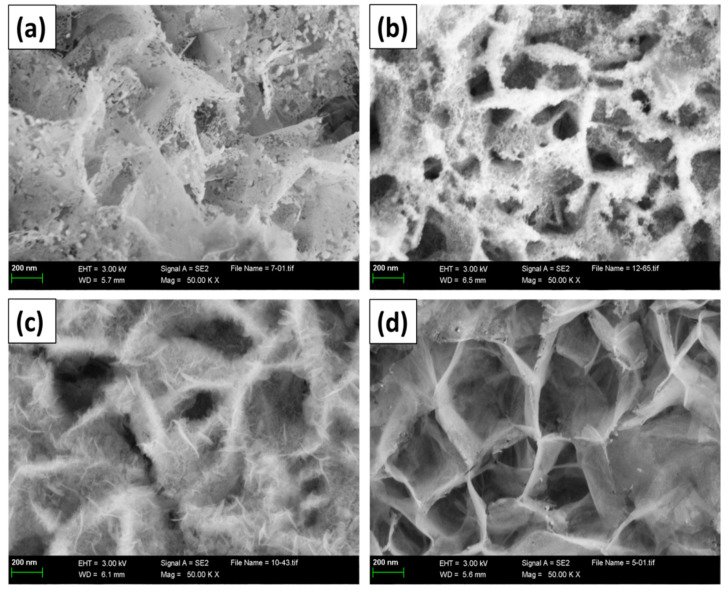
FESEM images of samples prepared at different MAHHTs: (**a**) 15 min, (**b**) 30 min, (**c**) 45 min, (**d**) 60 min; (300 × 300 DPI).

**Figure 7:**
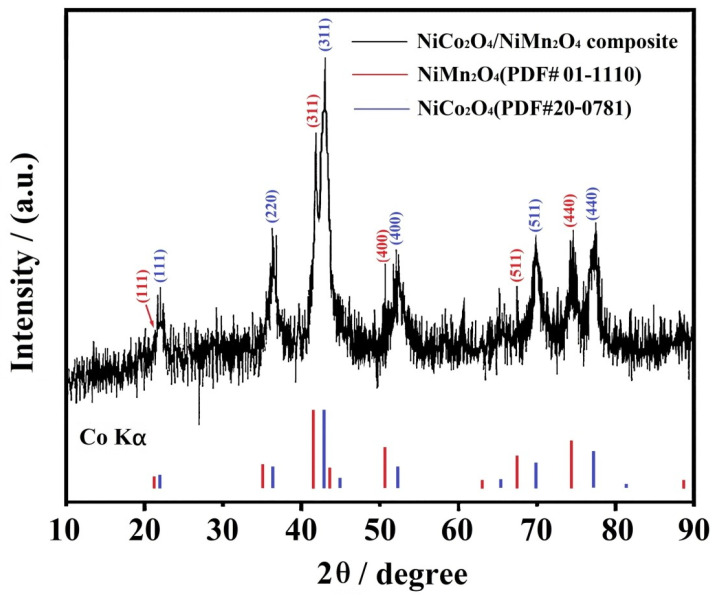
XRD pattern of the NiMn_2_O_4_@NiCo_2_O_4_ composite sample prepared at MAHHT of 30 min; (300 × 300 DPI).

**Figure 8:**
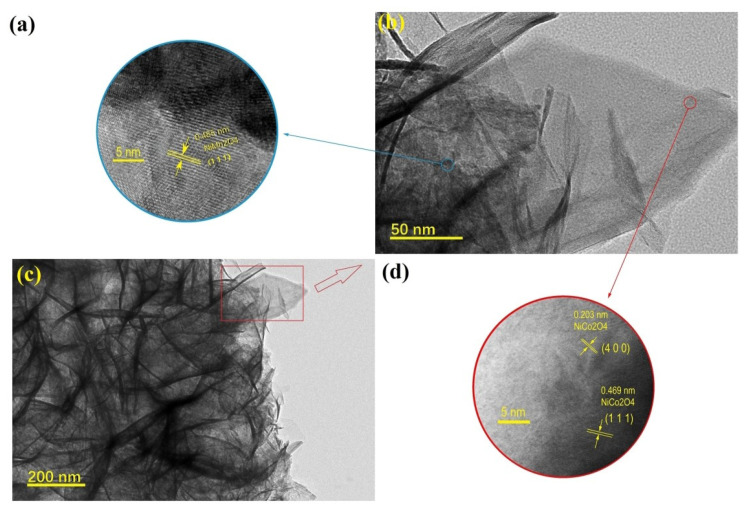
TEM images of the NiMn_2_O_4_@NiCo_2_O_4_ composite sample prepared at MAHHT of 30 min (**a**) Crystal plane analysis diagram of blue area (**b**) High-magnification mage (**c**) Low-magnification image (10 K) (**d**) Crystal plane analysis diagram of red area; (300 × 300 DPI).

**Figure 9:**
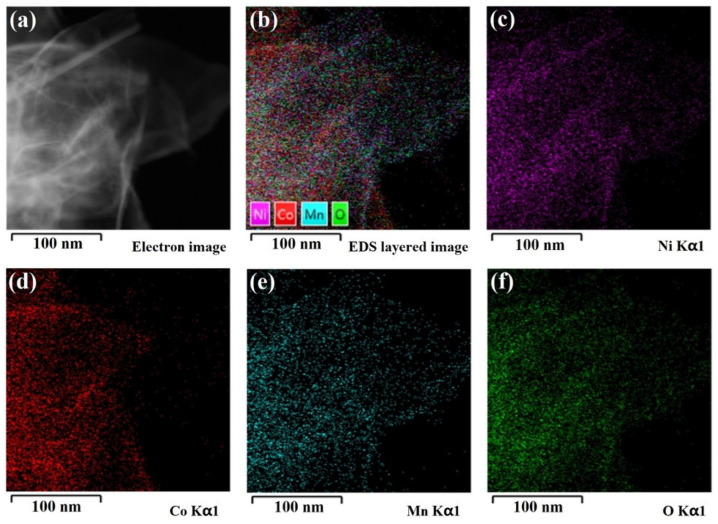
(**a**) SEM images of Figure 8b (**b**) EDS elemental mappings of Figure 8b: (**c**) Ni, (**d**) Co, (**e**) Mn, (**f**) O; (300 × 300 DPI).

**Figure 10:**
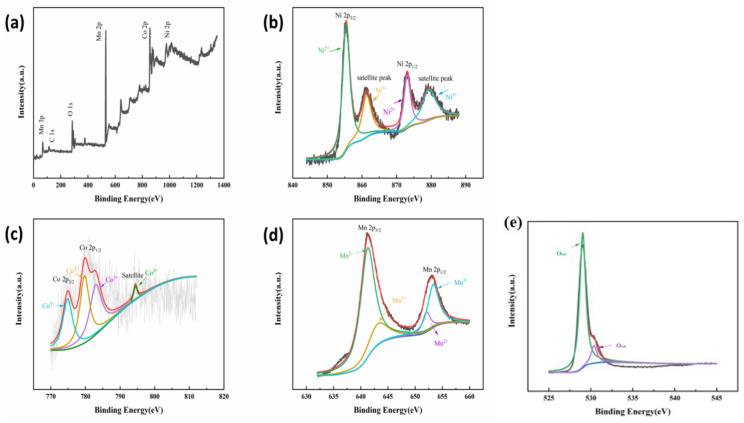
XPS of the NiMn_2_O_4_@NiCo_2_O_4_ composite sample prepared at MAHHT of 30 min (**a**) survey, (**b**) Ni 2p, (**c**) Co 2p, (**d**) Mn 2p and (**e**) O 1s; 213 × 180 (300 × 300 DPI).

**Figure 11:**
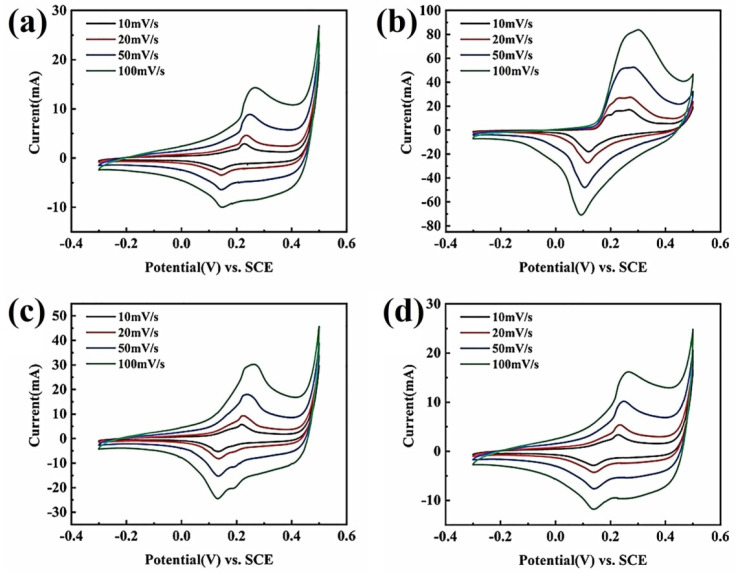
CV curves obtained at different scan rates (10, 20, 50, and 100 mV·s^−1^, respectively) of the NiMn_2_O_4_@NiCo_2_O_4_ composite samples prepared at different MAHHTs: (**a**) 15 min, (**b**) 30 min, (**c**) 45 min, (**d**) 60 min; (300 × 300 DPI).

**Figure 12:**
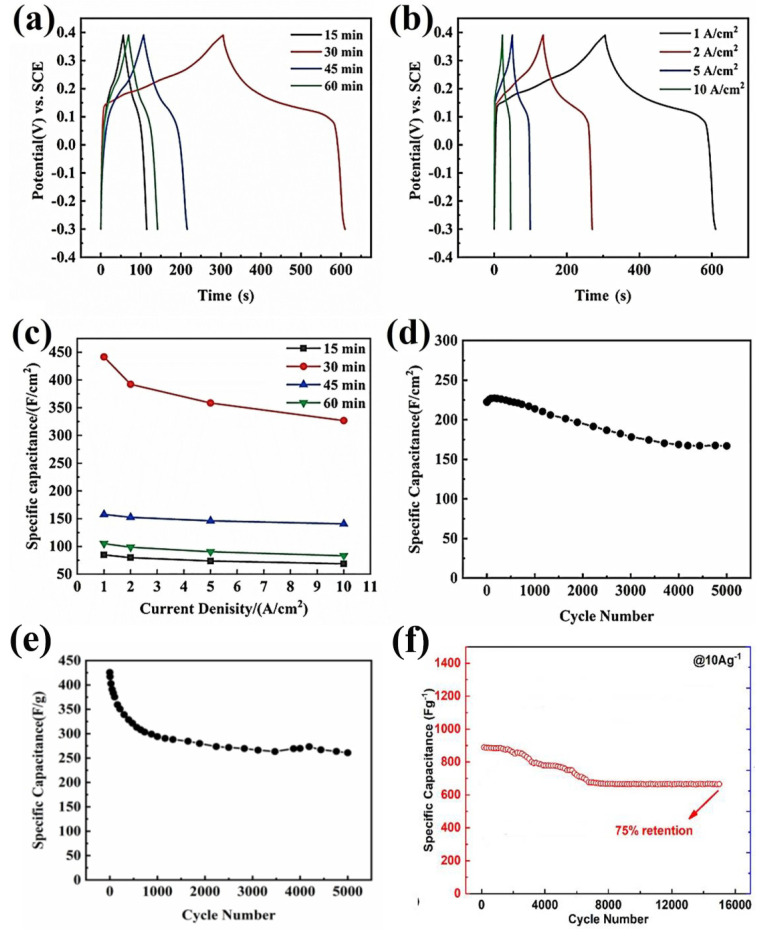
(**a**) GCD curves of the NiMn_2_O_4_@NiCo_2_O_4_ composite samples prepared at different MAHHTs (15, 30, 45, and 60 min, respectively) under the current density of 1 A·cm^−2^, (**b**) GCD curves of the NiMn_2_O_4_@NiCo_2_O_4_ composite sample prepared at MAHHT of 30 min under different current densities (1, 2, 5, and 10 A·cm^−2^, respectively), (**c**) specific capacitance as a function of current density, (**d**) cycling life curve after 5000 cycles at the current density of 15 A·cm^−2^ for the NiMn_2_O_4_@NiCo_2_O_4_ composite sample prepared at MAHHT of 30 min, (**e**) cycling stability of NiMn_2_O_4_@NF at 10 A/g for 5000 cycles, (**f**) cycling stability of NiCo_2_O_4_@NF at 10 A/g for 15,000 cycles; (300 × 300 DPI).

The SEM images of the NiMn_2_O_4_@NiCo_2_O_4_ nanocomposites prepared by the microwave-assisted hydrothermal method at different HT are shown in Figure 6. Compared with Figure 3, the NiMn_2_O_4_@NiCo_2_O_4_ composites exhibit distinct morphological differences while retaining the original NiCo_2_O_4_ nanosheet (NS) skeleton. At the MAHHT of 15 min (Figure 6a), the surface of the NiCo_2_O_4_ nanosheets exhibits highly three-dimensional wrinkles, resembling a mesh-like structure with numerous thin flakes growing vertically on the NiCo_2_O_4_ nanosheets. Moreover, several NPs’ (marked by red circles in Figure 6) surface coverage on NiCo_2_O_4_ NSs can also be observed, which are illustrated by Figure 1 and proved to be NiMn_2_O_4_ by following XRD (Figure 7), HRTEM (Figure 8), EDS (Figure 9) and XPS (Figure 10) analyses. When the MAHHT is increased to 30 min (Figure 6b), the NiCo_2_O_4_ NSs are more uniformly covered by newly formed NiMn_2_O_4_ crystal grains, resulting in a more three-dimensional and thicker surface wrinkling, thereby increasing the specific surface area of the NiMn_2_O_4_@NiCo_2_O_4_. As the MAHHT further rises to 45 min (Figure 6c), the quantity of NiMn_2_O_4_ NPs on the NiCo_2_O_4_ NSs surface increases significantly, and partial nanoparticles gradually evolve into tiny flake-like structures. The wrinkles of NiCo_2_O_4_ NSs become thicker due to these newly grown tiny flakes, and even thick enough to contact with adjacent wrinkles. Thus, many gaps are filled by these NiMn_2_O_4_ tiny flakes interlacing each other, resulting in a certain degree of decrement for the specific surface area of the NiMn_2_O_4_@NiCo_2_O_4_ composite. When the MAHHT is further increased to 60 min (Figure 6d), the NiMn_2_O_4_ tiny flakes grow further, making wrinkles of NiCo_2_O_4_ NSs thicker and thicker, and consequently, the specific surface area of the composite continues to decline. In summary, with MAHHT increasing from 15 to 30, 45 and 60 min successively, the specific surface area of the NiMn_2_O_4_@NiCo_2_O_4_ composite increases initially (with MAHHT from 15 to 30 min) and then decreases. The trend for the specific surface area of the composite can be obviously derived from Figure 6. Combined with the subsequent electrochemical performance analysis (Figure 11 and Figure 12), the variation trend of specific capacitance with MAHHT (Figure 12c) is highly consistent with the change in specific surface area. Accordingly, the composite fabricated at 30 min delivers the optimal electrochemical performance, with a maximum areal-specific capacitance of 441.56 F·cm^−2^ at a current density of 1 A·cm^−2^ among all prepared samples.

Figure 7 shows the XRD pattern of the NiMn_2_O_4_@NiCo_2_O_4_ composite prepared at MAHHT of 30 min. It can be clearly seen that the diffraction peaks are indexed at 2θ values of 22.06, 36.45, 43.06, 52.28, 69.85 and 77.32 degree, corresponding to the (111), (220), (311), (400), (511) and (440) crystal planes of NiCo_2_O_4_ (PDF#20-0781), respectively. Similarly, the diffraction peaks indexed at 2θ values of 21.36, 41.63, 50.62, 67.37 and 74.46 degree, correspond to the (111), (311), (400), (511) and (440) crystal planes of NiMn_2_O_4_ (PDF#01-1110), respectively. These XRD analysis results reveal that NiCo_2_O_4_ and NiMn_2_O_4_ have been successfully synthesized on the surface of 3D framework of NF substrate. Transmission electron microscopy (TEM) was used to further study the micromorphology of the composite prepared at MAHHT of 30 min. The low-magnification TEM images (Figure 8b,c) clearly reveal the retained tulle-like nanosheet architecture of the composite, which is consistent with the SEM observations. The HRTEM images (Figure 8a,d) display clear and continuous lattice fringes, demonstrating the high crystallinity of the as-prepared material. Specifically, the measured lattice spacing of 0.485 nm in Figure 8a matches well with the (111) crystal plane of NiMn_2_O_4_. The lattice spacing of the marked parts in Figure 8d is 0.203 and 0.469 nm respectively, which corresponds to the (400) and (111) crystal plane of NiCo_2_O_4_. As illustrated in Figure 9, Ni, Co, Mn, and O elements are uniformly distributed throughout the material, which further corroborates the successful growth of NiMn_2_O_4_ nanoparticles on the NiCo_2_O_4_ tulle-like skeleton, in good agreement with the XRD and HRTEM results. The surface composition and chemical valence states of the NiMn_2_O_4_@NiCo_2_O_4_ composite are gained by XPS analysis, which was measured on the sample prepared at MAHHT of 30 min, as shown in Figure 10. The survey spectrum of the sample (Figure 10a) confirms the presence and the binding energy values of Ni, Mn, Co, O, and C. The Ni 2p emission spectrum in Figure 10b displays two main spin–orbit peaks for 2p_3/2_ and 2p_1/2_ and two satellite peaks, suggesting the existence of both Ni^2+^ and Ni^3+^ species. The peaks at 855.4 and 873.0 eV are ascribed to divalent Ni^2+^, while those at 861.3 and 879.3 eV are assigned to trivalent Ni^3+^ [24,25]. Similarly, the Co 2p high-resolution spectrum (Figure 10c) shows two spin–orbit peaks of Co 2p_3/2_ and Co 2p_1/2_ and satellite peaks. The characteristic signals of the spectrum are relatively weak, which may be due to a large number of NiMn_2_O_4_ grains covering the surface of NiCo_2_O_4_ tulle-like NSs, resulting in signal interference with the characteristic peaks of Co. The Co 2p emission spectrum (Figure 10c) is fitted with two kinds of Co species. The fitting peaks at 774.9 eV and 783.0 eV are corresponding to Co^3+^, and the two peaks at 779.7 eV and 794.4 eV are attributed to Co^2+^ [26]. Figure 10d displays the high-resolution spectrum of Mn 2p. From the spectrum, it can be seen that there exist two kinds of Mn species corresponding to Mn 2p_1/2_ (Mn^2+)^ and Mn 2p_3/2_ (Mn^3+)^ in oxide form of NiMn_2_O_4_, respectively. The fitting peaks at 641.3 and 652.1 eV correspond to Mn^2+^, and those at 643.2 and 653.3 eV are attributed to Mn^3+^ [27,28]. The signal peaks of lattice oxygen (O_latt_) and adsorbed oxygen species (O_ads_) were detected at 529.9 eV and 531.3 eV, respectively. These findings strongly testify the formation of NiMn_2_O_4_@NiCo_2_O_4_ composite, which corresponds to the XRD (Figure 7), HRTEM (Figure 8), and EDS (Figure 9) results. Moreover, the multiple valence states of Ni, Co and Mn in the NiMn_2_O_4_@NiCo_2_O_4_ composite are critical for achieving high specific capacitance in charge storage for pseudocapacitors. It is well-known that pseudocapacitors store charges only in the first few nanometers from the surface. Benefiting from the unique hierarchical structure of NiMn_2_O_4_ nanoparticles decorated on NiCo_2_O_4_ nanosheets, the NiMn_2_O_4_@NiCo_2_O_4_ composite possesses a large specific surface area and abundant porous structures. These structural features provide sufficient electrochemical active sites and favorable ion-transport channels, thereby effectively improving the overall electrochemical performance of the supercapacitor electrode.

Figure 11 shows the CV curves of NiMn_2_O_4_@NiCo_2_O_4_ nanocomposite samples prepared at different MAHHT, which were obtained at different voltage scan rates including 10, 20, 50, and 100 mV·s^−1^ within a potential range of −0.3~0.5 V. All curves have obvious redox peaks and nearly symmetrical shapes with distinct voltage plateaus, further demonstrating their reversible faradaic reactions and pseudocapacitive performance. For the same sample, as the voltage scan rate increases, there is a significant increase in the redox peak absolute value and the shape of the curve does not change much, showing a comparatively small resistance for the NiMn_2_O_4_@NiCo_2_O_4_ electrode, and a rapid redox reaction occurred between the electrolyte and the electrode at the interface [27]. Among all samples prepared at different MAHHTs, the redox peak of the sample prepared at an MAHHT of 30 min (Figure 11b) is the most prominent, and the absolute integral area of its CV curve is the largest for the same voltage scan rate, reflecting its better reversibility and magnification than the other samples.

Figure 12a shows the GCD curves of the samples prepared at different MAHHTs under the current density of 1 A·cm^−2^. It can be seen that each curve has a charging and discharging platform that can represent the characteristics of pseudocapacitance, and the single charging and discharging time of the sample prepared at MAHHT of 30 min is the longest amongst all samples. The GCD curves (Figure 12b) of the sample prepared at an MAHHT of 30 min were performed under different current densities (1, 2, 5, and 10 A·cm^−2^, respectively). Figure 12b shows that the single charging and discharging time decreases with the current density increasing. Based on the GCD curves of each sample under different current densities, the corresponding specific capacitance was calculated by Equation (2), and Figure 12c was obtained.

Like the single charge and discharge time, the calculated specific capacitance gradually decreases as the current density increases. As shown in Figure 12c, the sample prepared at an MAHHT of 30 min has the highest specific capacitance at different current densities among all samples; i.e., the specific capacitance is 441.56, 392.27, 358.47, and 326.77 F·cm^−2^, respectively, when the current density is 1, 2, 5, and 10 A·cm^−2^, on the basis of analyzing the CV curves. In order to further study the cycling stability of the sample prepared at an MAHHT of 30 min, 5000 charge–discharge cycles were performed at a current density of 15 A·cm^−2^, and the cycle life curve (shown in Figure 12d) was obtained after calculation. It is found that the specific capacitance can still retain 75% of the initial value after 5000 cycles, which indicates that the sample has a good long-term cycling stability as an electrode. Figure 12e and Figure 12f illustrate the cycling stability of NiMn_2_O_4_@NF and NiCo_2_O_4_@NF at 10 A/g, respectively. The capacity retention rate for the former is 61% while that value is ~75% for the latter after 5000 cycles. The value retains 75% after 5000 cycles at 15 A cm^−2^ when NiMn_2_O_4_ and NiCo_2_O_4_ are combined together. Therefore, the synergistic effect between the two materials enhances their cycling stability.

## 4. Conclusions

In this work, a binder-free NiMn_2_O_4_@NiCo_2_O_4_ composite electrode for high-performance supercapacitors was successfully fabricated on nickel foam via a two-step microwave-assisted hydrothermal (MAH) strategy combined with post-annealing treatment, without introducing any conductive additives or binders. The experimental results demonstrate that the composite prepared at an MAH holding time of 30 min possesses a unique porous tulle-like architecture, where NiCo_2_O_4_ nanosheets uniformly anchor and wrap NiMn_2_O_4_ nanoparticles/nanoflakes, exhibiting the optimal comprehensive electrochemical performance among all samples. The superior electrochemical behavior is mainly attributed to the synergistic coupling effect between NiCo_2_O_4_ and NiMn_2_O_4_, as well as the advantageous structural characteristics of the composite, including abundant porous structure, suitable pore size distribution, and the largest specific surface area. These properties effectively prevent structural degradation and collapse during long-term electrochemical cycling, provide numerous electrochemically active sites, and construct efficient transmission pathways for electrons and electrolyte ions. Electrochemical tests reveal that the optimized NiMn_2_O_4_@NiCo_2_O_4_ electrode delivers an outstanding areal-specific capacitance of 441.56 F·cm^−2^ at 1 A·cm^−2^, together with satisfactory long-term cycling stability, maintaining 75% of its initial capacitance after 5000 continuous charge–discharge cycles at 15 15 A·cm^−2^. This work confirms that the rational design and fabrication of NiMn_2_O_4_@NiCo_2_O_4_ hierarchical composites is a feasible and effective strategy to develop high-performance electrode materials, which endows the material with great application potential for advanced supercapacitor devices.

## Data Availability

The original contributions presented in this study are included in the article. Further inquiries can be directed to the corresponding authors.
